# Should Atypical and Non-Representative Studies Such as NutriNet Santé Be Used to Drive Public Health Policy?

**DOI:** 10.3390/nu17162581

**Published:** 2025-08-08

**Authors:** Adam Drewnowski, Victor L. Fulgoni

**Affiliations:** 1 Center for Public Health Nutrition, University of Washington, Seattle, WA 98195, USA; 2 Nutrition Impact LLC, Battle Creek, MI 49014, USA; vic3rd@aol.com

**Keywords:** NutriNet Santé, National Health and Nutrition Examination Survey (NHANES 2011-18), low calorie sweeteners (LCS), aspartame, diet quality, Healthy Eating Index 2020, LCS consumers

## Abstract

**Background**: Findings from the NutriNet-Santé studies have been used to drive public health policy in France, the European Union, and globally. The fact that NutriNet-Santé studies are not generalizable is a matter of concern. **Objectives**: We aimed to compare the characteristics and diet quality of consumers and non-consumers of low-calorie sweeteners (LCS) within the National Health and Nutrition Examination Survey (NHANES) sample to published within-cohort findings from NutriNet Santé. **Methods**: Dietary intake data for the US from two 24 h dietary recalls in 4 cycles of the NHANES 2011-18 study (*n* = 17,252) were used to identify LCS consumers and non-consumers. **Results**: LCS consumers in NHANES were more likely to be overweight, were of higher education and incomes, and had lower intakes of added sugar and higher HEI 2020 diet quality scores compared to LCS non-consumers. Based on published reports, higher LCS consumers in NutriNet Santé were normal weight, did not differ in education, did not consume less added sugar, and had lower quality diets overall. Whereas LCS consumers in NHANES were *less* likely to be current smokers, higher LCS consumers in NutriNet Santé were *more* likely to be current smokers. Based on published estimates, mean aspartame intake in NutriNet Santé was only 3 mg/day (0.045 mg/kg/day) for lower consumers and 47 mg/day (0.71 mg/kg/day) for higher consumers. **Conclusions**: Minimal LCS exposure and likely floor effects can be sources of statistical biases in studies of diet and health. NutriNet Santé is a large volunteer cohort of thin, educated, weight conscious French women who diet and smoke. Extreme caution is warranted when findings from atypical and non-representative samples are used to support policies in global public health.

## 1. Introduction

The NutriNet-Santé study in France is an ongoing web-based cohort study [1] designed to explore associations between diets and health [2,3,4,5]. Beginning in 2009, self-referred volunteers with internet access were recruited through extensive television, radio, print, and social media campaigns. Data continue to be collected online [1]. Questionnaires ask about socio-demographics, lifestyles, height and weight, health status, physical activity, and diet [1,2,3,4,5,6,7].

Because of its large sample size, the NutriNet-Santé cohort has been billed at times as a population-based study [2,6]. That is not quite correct. As is the case with most volunteer cohorts, NutriNet-Santé participants were of higher socioeconomic status than the general population, were specifically interested in diet and lifestyle, and were predominantly women [2,3,4,5]. Those limitations are dutifully listed in published papers [2,3,4,5]. There is repeated caution that the sample is not representative, causality cannot be established, and the results ought not to be generalized to French adults as a whole [2,3].

This has not stopped NutriNet-Santé findings from being used to support public health policies in France, the European Union, and globally. NutriNet-Santé data have been used by French, European, and international health agencies to link dietary habits with self-reported health outcomes. The World Health Organization’s (WHO) recent reports on cancer hazards took NutriNet-Santé findings on low-calorie sweeteners (LCS) and cancer risk into account [8,9]. More recently [10], NutriNet-Santé findings were used to justify the need for strong efforts to further transition to the EAT-Lancet planetary health diet.

Representativeness is one critical element in population-based studies. However, the impact of selection bias on the observed relations between diet and health is sometimes hard to judge [11]. Data from a non-representative sample may still have internal validity, meaning that the relationship between exposure and outcome within the sample may still hold, even if the findings may not apply to other populations. Unequally distributed variables are generally adjusted for in regression analyses. For example, Debras et al. [2] adjusted the main analyses for age, sex, education, physical activity, smoking status, body mass index [BMI], height, weight gain, personal and family medical history, number of 24 h diet records, and baseline intakes of energy, alcohol, sodium, saturated fatty acids, fiber, total sugar, fruit and vegetables, whole-grain foods, and dairy. Data on incomes and race/ethnicity) could not be adjusted for, since such data are not collected in France.

LCS in beverages and foods are used primarily for the purpose of weight control [12,13,14]. The accepted mechanism is a reduction in added sugar intakes, mainly from lower consumption of sugar-sweetened beverages. The present goal was to compare the characteristics and behaviors of LCS consumers and non-consumers within each study sample. Data on characteristics and dietary behaviors of LCS consumers and non-consumers in the NutriNet-Santé cohort were published by Debras et al. [2]. Those data were compared to parallel within-sample findings in the nationally representative National Health and Nutrition Survey (NHANES 2011-18) in the US. The present focus was on the characteristics of LCS consumers and non-consumers and the directionality of any observed effects.

## 2. Materials and Methods

### 2.1. National Health and Nutrition Examination Survey NHANES 2011-18

The National Health and Nutrition Examination Survey (NHANES) [15,16] is a nationally representative population-based study of diets and health. Despite its cross-sectional nature, NHANES dietary surveillance is the cornerstone of food and nutrition policies in the US [17].

NHANES 2011-18 cycles provided data for 17,252 participants (8372 males and 8880 females) aged >19 y who completed valid 24 h dietary recalls. Demographic data were obtained by self-report. Gender was defined as male or female. Education level was defined as high school or less (12 y), some college (12–16 y), and college graduate (>16 y). Household income was assessed using the poverty-to-income ratio (PIR) with cut points set at: <1.35, 1.35 to 1.85, and >1.85. Race/ethnicity was non-Hispanic White, non-Hispanic Black, Hispanic, and Asian. Smoking status was classified as current, former, or never smokers. The prevalence of diabetes diagnosis was obtained by self-report.

Measured body weight and height were used to determine body mass index (BMI = kg/m^2^). Participants were classified as underweight (<18.5 BMI), normal weight (18.5–25 BMI), overweight (25–30 BMI), and obese (>30 BMI). The Institutional Review Board (IRB) approvals for NHANES had been obtained by the National Center for Health Statistics (NCHS) [18]. NHANES data are publicly available [15].

### 2.2. Nutrient Composition Data

The dietary component of NHANES is known as the What We Eat in America Study [15,16]. Two 24 h dietary recalls are collected during in-person visits and by phone [16]. The multi-pass method probes for foods and beverages consumed over the previous 24 h [16], recording both times and eating occasions. The present analyses used the mean of two 24 h dietary recalls that were available for about 90% of the sample. All NHANES dietary recalls are publicly available [16].

The Food and Nutrient Database for Dietary Studies (FNDDS) is used to calculate the energy and nutrient content of NHANES diets. The FNDDS is maintained by the US Department of Agriculture (USDA) and is available on FoodData Central [19]. Since the FNDDS does not automatically call out beverages and foods containing low-calorie sweeteners (LCS), a customized coding approach was required. Following published methods [20,21,22], all foods in the FNDDS file were examined for the presence of LCS by using the food description, energy density (kcal/100 g), and total and added sugars content per g/100 g and per mean consumption amount. Added sugars intakes and amounts of MyPlate food groups were obtained using the Food Patterns Equivalent Database. The food categories and their 8-digit WWEIA codes are provided in Supplementary Table S1.

Less than 2% (*n* = 144) of all FNDDS foods were identified as containing LCS. The most common LCS foods were soft drinks (cola-type or fruit flavored) that were further described as “sugar-free”, “low-calorie’, or “diet” [23]. Also included were teas pre-sweetened with LCS. The FNDDS also listed a limited number of solid foods (diet yogurt, ice cream, grain-based desserts, and candies) and tabletop LCS [20,21,22]. For tabletop LCS powder products used to sweeten beverages, coffee, or tea, the weight of the beverage to which the LCSs were added was set at 170 g (grams of 6 oz coffee) plus the negligible weight of the powder itself. Those data are consistent with recent calculations showing that approximately 95.8% of aspartame in the US came from soft drinks and other beverages (including coffee and tea), followed by sweetener packets, yogurt, ice cream, snacks, mints, and chewing gum [24].

Estimating current aspartame intakes in mg/day was more problematic. USDA nutrient composition data typically list aspartame content of diet beverages at around 50 mg/100 g, equivalent to 180 mg for 12 oz serving. That is the amount that is still listed by most agencies, including the World Health Organization (WHO) [8]. However, diet beverages in the US are now sweetened with blends of aspartame and acesulfame K, whereas some are sweetened with sucralose or stevia. The FNDDS database does not contain brand names. Potential ranges of aspartame intake were estimated based on the amounts of diet beverages consumed.

### 2.3. Diet Quality Metrics: Healthy Eating Index HEI 2020

Dietary data, including amounts of MyPlate food groups (in oz or cup eq.), were used to calculate Healthy Eating Index (HEI 2020) scores, a measure of compliance with the Dietary Guidelines for Americans [25]. The HEI 2020 is a 100-point scale, composed of 13 sub-scores that include added sugars, sodium, whole grains, saturated fat, as well as fat ratios.

### 2.4. Plan of Analysis

Individuals who consumed any amount of LCS food or LCS beverage on either day of 24 h recalls were identified as LCS consumers. Consumption estimates were obtained for the total weights of beverages, foods, and tabletop LCS expressed in g/day.

LCS consumers and non-consumers were then compared on demographics, body weight, and dietary variables. The significance of differences was tested using regression models adjusted for age, gender, race/ethnicity, education level, physical activity level, smoking current status, alcohol use, and body mass index.

Regression analyses adjusted for the complex sampling plan of NHANES. The NHANES specific variables were sample weight (WTINT2YR), strata (SDMVDTRA), and Primary Sampling Unit (SDMVPSU). Main analyses compared LCS consumers and LCS non-consumers within the NHANES sample. Analyses used the SAS9.4 statistical package. Statistical significance was set at *p* < 0.05.

## 3. Results

### 3.1. Socio-Demographic Profiles and LCS Use

Table 1 summarizes the main socio-demographic characteristics for the NHANES and NutriNet-Santé participants. The NHANES was a nationally representative sample of the US population. Each NHANES cycle is said to be representative of 190–200 million US adults. NHANES sample characteristics are well described. As a comparison, the NutriNet-Santé cohort was 78.5% female, with a BMI of 23.7 (normal weight), highly educated (65.1% had 2 y of college), with a very low prevalence of diabetes (1.48%) and high reported prevalence of LCS use (36.9%) [2].

The main analyses compared LCS consumers and non-consumers within each study sample. LCS use is generally associated with higher education and incomes [17,18,19,20]. In NHANES, higher education was associated with higher LCS use (28.4% vs. 37.5%). In NutriNet-Santé, the effect of higher education was minimal (64.9% vs. 65.1%). In NHANES, higher incomes (as poverty-to-income ratios (PIRs)) were associated with higher LCS use (52.3% vs. 75.1%). NHANES LCS consumers were more likely to be non-Hispanic whites (75.1%) and less likely to identify as Hispanic, Black, or Asian. NutriNet Santé does not provide data on incomes or race/ethnicity. Those data are generally not collected in France.

LCS use is generally associated with a higher prevalence of obesity and type 2 diabetes. NHANES LCS consumers were more likely to be obese than non-consumers (45.82% vs. 35.70%) and were more likely to report a diagnosis of diabetes (19.81% vs. 8.89%). BMI values were 30.38 for LCS consumers and 28.74 for non-consumers. BMI values in NutriNet Sante were 23.3 (normal weight) for non-consumers and 24.96 (also normal weight) for higher LCS consumers. The prevalence of type 2 diabetes was 1.0% among non-consumers and 2.8% among consumers. The reported prevalence of dieting to lose weight among higher LCS consumers in NutriNet Sante was 32%.

LCS use is generally associated with less current smoking. NHANES LCS consumers less likely to be current smokers than non-consumers (19.8% vs. 13.7%.). Higher LCS consumers in NutriNet Santé were *more* likely to be current smokers than non-consumers (17.2% vs. 20.3%).

### 3.2. LCS Use and Compliance with the Dietary Guidelines for Americans

LCS use is generally associated with lower consumption of regular sugar sweetened beverages and with lower intakes of added sugars. NHANES LCS consumers had lower intakes of added sugar (53.3 g/day or 10.5% of energy) than LCS non-consumers (71.4 g/day or 13.5% energy). By contrast, mean added sugar intakes in NutriNet Santé were not greatly reduced by LCS use [2]. Reported intakes of added sugars were 38.4 g/day for non-consumers and 37.8 g/day for higher LCS consumers. Mean percent of energy from added sugar was 7.9% for non-consumers and 7.9% for higher LCS consumers.

LCS use is generally associated with higher quality diets, given the association of LCS use with higher education and incomes. The Healthy Eating Index HEI-2020 [24] provides a measure of compliance with the USDA Dietary Guidelines for American and typically serves as an overall measure of diet quality. HEI 2020 sub-scores track the consumption of fruits, vegetables, dairy, and whole grains along with sodium, sugar, and saturated fat [2].

**Figure 1:**
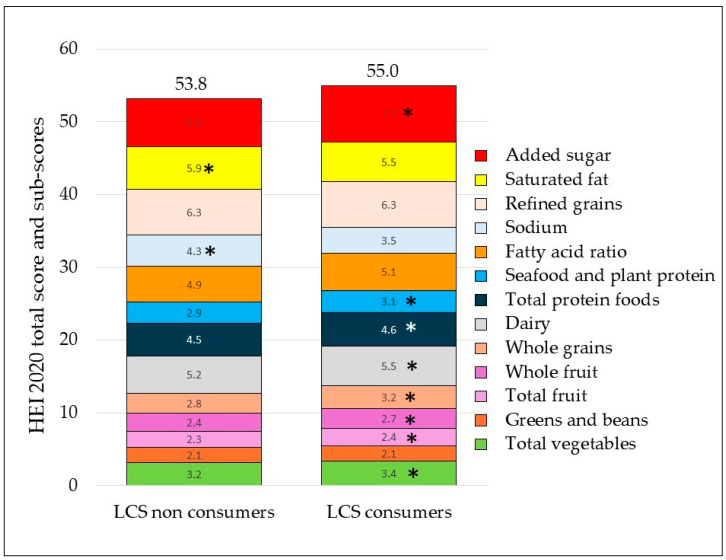
HEI 2020 total scores and sub-scores for LCS consumers and non-consumers in the NHANES 2011-18 dataset. Asterisks denote significant differences between sub-scores.

Figure 1 shows that LCS consumers had higher HEI 2020 total scores and more higher sub-scores than non-consumers. LCS consumers had higher sub-scores on total vegetables (*p* < 0.001), whole fruit (*p* < 0.0001), total fruit (*p* < 0.05), whole grains (*p* < 0.0001), dairy (*p* < 0.5), and seafood and plant protein (*p* < 0.005). Added sugar sub-scores were significantly higher (*p* < 0.0001), indicating that added sugar intakes were greatly reduced with LCS use. There were no significant differences for greens and beans, refined grains, and the fatty acid ratio sub-scores. Not all aspects of LCS users’ diets were better. Lower HEI 2020 sub-scores for LCS consumers for sodium and saturated fat indicated higher consumption for both (*p* < 0.0001).

Higher LCS users in the NutriNet-Santé study reported diets that differed in some important ways from those of LCS users in NHANES. Surprisingly, high LCS consumers actually drank significantly more regular sugar-sweetened beverages than did LCS non-consumers (57.9 + 0.9 mL/day vs. 42.8 + 0.4 mL/day). Compared to LCS non-consumers, higher LCS consumers had diets with significantly less fruit and vegetables per day (397.5 + 1.68 g/day vs. 409.0 + 0.9 g/day), and less whole grain foods (31.2 + 0.3 g/day vs. 36.0 + 0.2 g/day). Their diets had significantly more dairy products (234.4 + 1.2 g/day vs. 183.6 + 0.6 g/day) but less saturated fat (31.9 + 0.1 g/day vs. 33.6 + 0.05 g/day). All the differences were reported (2) at *p* < 0.0001 level of significance.

### 3.3. Estimation of Aspartame Intake

**Figure 2:**
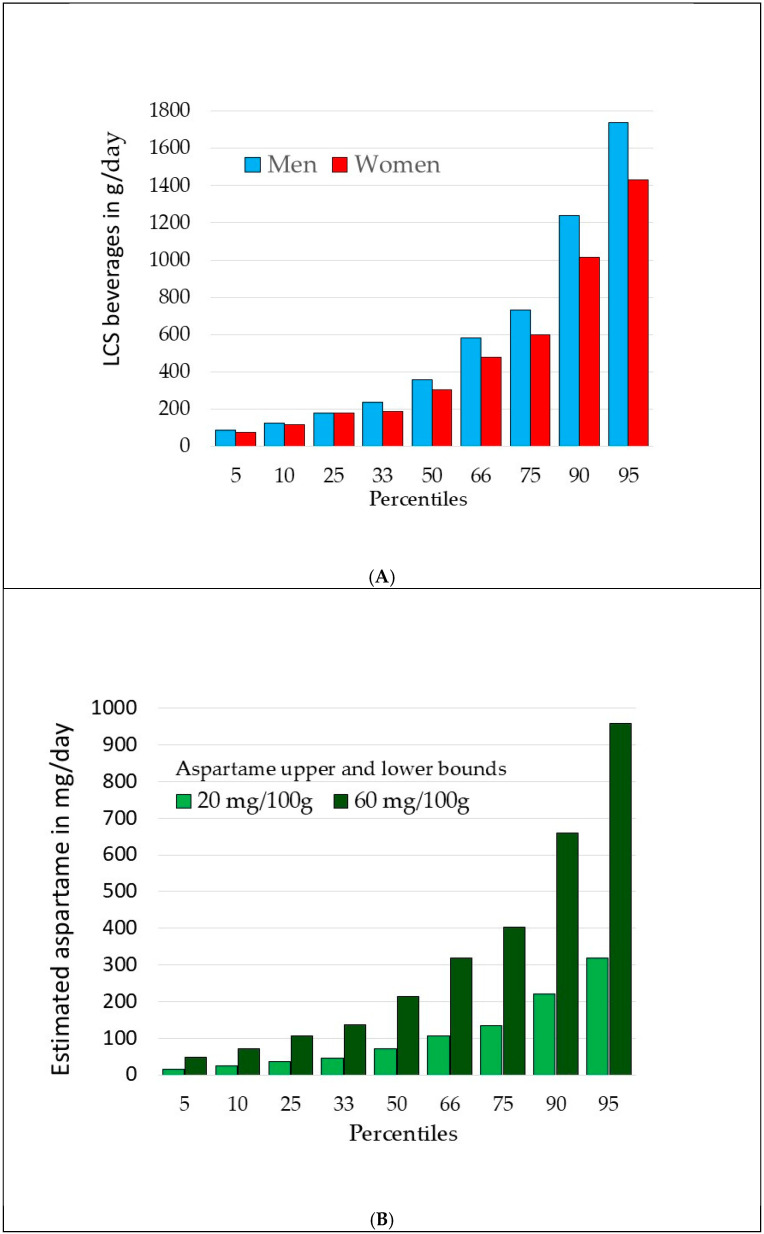
Percentile distribution of consumption of LCS-sweetened beverages by sex (**A**) and percentile distribution of theoretical upper and lower bounds for estimated aspartame intakes by adults aged 19+ y (**B**).

Most LCS in NHANES dietary intakes came from beverages (87%) rather than solid foods (12%). For the present analyses, tabletop LCS use was converted to beverage grams, assuming 170 g for a cup of tea or coffee. Figure 2A shows the percentile distribution of LCS beverage intakes among consumers aged 19+ y by sex. Median values were 304 g/day for women and 359 g/day for men. Mean values, weighted by the heavy consumers, were much higher: 477 g/day for women and 571 g/day for men.

Estimating aspartame intakes required some assumptions. The 24 h recalls in the NutriNet-Santé study collected data on brands and commercial names of low-calorie beverages (2) so that individual LCS could be identified. The NHANES database allowed us to do this for tabletop LCS but not for low-calorie beverages. We therefore set theoretical lower and upper limits for aspartame intakes. According to the FDA and the WHO, each 12 oz can of diet soda contains 180 mg of aspartame or 50 mg/100 g. However, aspartame is increasingly used in LCS blends, most often combined with acesulfame-K. We set the lower and upper bounds for aspartame in LCS beverages at 20 mg and 60 mg per 100 g, respectively, bracketing the FDA estimate.

Figure 2B shows the lower and upper bounds for aspartame based on LCS beverage amounts. Assuming that all the LCS beverages consumed by NHANES participants were sweetened with aspartame (which is not the case), that would give us median consumption of 71 mg/day to 213 mg/day. At higher levels (75th percentile), theoretical aspartame exposure would be from 134 to 403 mg/day.

The Acceptable Daily Intake (ADI) for aspartame is 50 mg/kg/day which is 3500 mg/day for a 70 kg individual. It is important to realize that mean aspartame intakes for lower users in the NutriNet-Santé cohort were only 3 mg/day (0.045 mg/kg/day), that is to say 1000 times less. Mean intake for higher users (above 80th percentile) was 47.4 mg/day (0.71 mg/kg/day).

## 4. Discussion

The demographic profiles of NutriNet-Santé LCS consumers have been described before [1,2,3,4,5,6,7]. The present analyses compared LCS users and non-users within the NHANES 2011-18 and NutriNet-Santé study samples, the latter based on published data from Debras et al. [2]. There were several discrepancies in the patterns of LCS use and in the directionality of the observed effects [1,2,3,4,5,6,7].

First, the NutriNet-Santé cohort was of uniformly high education with little socioeconomic diversity. In most other studies, LCS use has been associated with higher education and incomes [20,21,22]. In NHANES, higher education was associated with a 9% increase in LCS use. In NutriNet Santé, the increase was only 0.2% [2]. In NHANES, LCS use was highest among non-Hispanic Whites. Incomes and race/ethnicity are among the major factors influencing any links between diet and health. Those crucial data were not available in NutriNet Santé.

Second, in most past studies, LCS use has been associated with less current smoking. NHANES [20], the Nurses’ Health Study, and the Health Professionals Follow Up Study [25] are all consistent on that important point. By contrast, LCS consumers in NutriNet Santé were more likely to be current smokers. Smoking is a well-known method to control body weight and has been associated with cancer risk.

Third, LCS use has been associated (in cross-sectional studies) with a higher prevalence of obesity and diabetes [22,23]. Those basic relations were observed in NHANES studies, the Nurses’ Health Study, and the Health Professionals Follow Up Study [26]. In NutriNet Santé, both LCS consumers and non-consumers were of normal weight (BMI < 25) [2]. Even so, LCS use was prevalent, with one-third of the sample reported dieting to lose weight.

Fourth, LCS use is invariably associated with lower observed intakes of added sugar and lower intakes of regular sweetened soft drinks. Indeed, lower observed intakes of added sugar normally serve to validate LCS use. The observed reduction in added sugar was as much as 18 g/day in NHANES but only 0.5 g/day in NutriNet Santé. Percent energy from added sugars in NutriNet Santé was 7.85% among LCS users and 7.88% among non-users [2].

Unexpectedly, higher LCS consumers in NutriNet Santé actually consumed more regular sugar-sweetened beverages than did non-consumers. The failure to find a significant reduction in added sugar intake and an increase in sugar-sweetened beverages raises questions about data quality and accurate estimates of LCS exposure.

Finally, here and in past studies, LCS use has been associated with higher-quality diets [21,27]. Past studies of LCS users [26] have reported diets with more vegetables and fruit, more diet beverages, and sharply lower consumption of regular sugar-sweetened beverages by both women and men. In the present NHANES sample, LCS consumers had higher HEI 2020 scores overall, albeit the increase was small. HEI 2020 sub-scores were higher for total vegetables, whole fruit, total fruit, whole grains, dairy, seafood, and plant proteins.

By contrast, higher LCS consumers in NutriNet Santé reported diets with less fruits and vegetables, less whole grains, and less fiber. In NHANES, LCS consumers had higher intakes of sodium and saturated fat. In NutriNet Santé, higher LCS consumers had higher intakes of dairy but lower intakes of saturated fat. Dairy is a major source of saturated fat in France. These opposing trends in diet quality among LCS users and non-users cannot be readily explained.

It needs to be pointed out that the estimated aspartame intakes in NutriNet Santé were extremely low. Many calculations assume that each 8 oz serving of a diet beverage contains 180 mg of aspartame, as was the case some years ago. For example, Schermerhorn et al. [26] estimated aspartame intakes among higher users at 248 mg/day for women and 268 mg/day for men. However, diet beverages are now sweetened with LCS blends, sucralose, and stevia, and estimating exposure is a challenge [24]. The present estimate for the median intake of aspartame based on potential upper and lower bounds was from 71 mg/day to 213 mg/day.

By contrast, mean aspartame intake for low users in the NutriNet-Santé sample was only 3 mg/day, equivalent to 0.045 mg/kg/day. The US FDA’s Acceptable Daily Intake (ADI) for aspartame is 50 mg/kg/day. In other words, aspartame exposure in the Debras et al. [2] study was estimated at 1/1000 (0.1%) of the FDA ADI. For higher consumers, representing 18% of the sample, the estimate was 0.71 mg/kg/day or approximately 1.5% of the FDA ADI. Those values suggest a limited and narrow range of exposure and a high likelihood of floor effects.

Debras et al. [2] have recognized the low level of LCS exposure in the NutriNet-Santé cohort, suggesting that the link to cancer risk may have been underestimated as a result. Another possibility is floor effects, a situation where most study participants have zero or close-to-zero LCS exposure. Floor effects are known to distort statistical models, leading potentially to false positives. The statistical noise may preclude any valid conclusions from NutriNet Santé about LCS exposure and cancer risk or any other health outcome. A recent analysis of the NHANES population with a much broader range of LCS exposure failed to find any relation between LCS and cancer risk [23].

Both studies had limitations. Both NHANES and NutriNet Sante were based on dietary self-reports that are subject to misreporting and recall bias. Cross-sectional studies cannot be used to establish temporality and causality. Some of the health variables were obtained by self-report, whereas others (height, weight) were measured in NHANES but not in NutriNet Santé. Residual confounding may have influenced associations due to dietary restraint, physical activity, or other unmeasured variables. While individual tabletop sweeteners could be identified, the exact composition of contemporary diet beverages cannot be assessed with precision.

Volunteer cohorts should not be dismissed altogether. In general, such cohorts are composed of motivated participants who are willing to follow protocols, complete dietary and health surveys, and remain with the study over long periods of time. Data from volunteer cohorts are valuable for hypothesis generation and may also have internal validity, where the relation between exposure and outcome still holds within the sample. The NutriNet-Santé study has produced data on diet quality trends [28] in France, mindful eating [29], and the typology of out-of-home eaters [30].

## 5. Conclusions

Nonetheless, based on the present analyses, we conclude that NutriNet Santé is an atypical cohort of thin, educated, weight-conscious French women who diet and smoke. Consumption of LCS was minimal, and the consumption of sugar-sweetened beverages actually increased with aspartame use. Added sugar intakes were not substantially reduced. We agree with the authors of [2] that causal links between LCS use and health outcomes cannot be established and residual confounding bias cannot be entirely ruled out. We also agree that the NutriNet-Santé findings may not be generalizable to the adult French population or to any population.

Why the atypical and non-representative NutriNet-Santé study findings continue to play a role in public policy dialog is a good question. In July 2023, the International Agency for Research on Cancer classified aspartame as “possibly carcinogenic to humans” (Group 2B), citing “limited evidence” from the large French NutriNet-Santé cohort [8,9]. The finding, disputed by the FDA, showed a 15% increase in cancer risk among the “highest consumers” of aspartame, including breast and obesity-related cancers [2].

One answer is that there are no studies to compare it to. The nationally representative French INCA 3 study released only last year is limited to about 5800 participants and is actually 14 years old [31]. The present suggestion is for the French ANSES agency to launch a nationally representative INCA 4 that includes some clinical health outcomes. In the meantime, extreme caution should be used when using NutriNet-Santé findings to support any public health policy measures, whether in France or elsewhere.

## Figures and Tables

**Table 1:** LCS consumers and non-consumers in NHANES 2011-18 and in NutriNet-Santé studies by socio-demographic and health variables.

NHANES 2011-18 (Ages 19+)	Total	Non-Consumers	Consumers	*p* Value
Sample size, *n* (%)	17,253 (100)	12,085 (70.04)	5168 (29.95)	
Sex *n* (%)				
Male	8373 {48.5)	6030 (49.9)	2343 (45.33)	0.0000
Female	8880 (51.4)	6055 (50.1)	2825 (54.66)	
Educational level *n* (%)				
<12 y	6210 (36.02)	4660 (38.56)	1600 (30.96)	0.0000
<14 y	5615 (32.57)	3998 (33.08)	1630 (31.55)	0.2864
>14 y	5415 (31.41)	3426 (28.35)	1937 (37.49)	0.0000
Poverty to income ratio *n* (%)				
PIR < 1.35	3688 (23.39)	2941 (26.73)	8009 (16.82)	0.0000
PIR 1.35–1.85	1576 (10.00)	1221 (11.00)	3831 (8.04)	0.0001
PIR > 1.85	10,496 (66.60)	6848 (62.26)	3578 (75.13)	0.0000
Race/ethnicity				
NH White	11,206 (64.95)	7233 (59.85)	3881 (75.09)	0.0000
Hispanic	2555 (14.81)	1994 (16.50)	5917 (11.45)	0.0000
NH Black	1963 (11.38)	1622 (13.42)	379 (7.33)	0.0000
Obese *n* (%)	6657 (39.09)	4273 (35.70)	2337 (45.82)	0.0000
Diabetes (told) *n* (%)	2162 (12.54)	1074 (8.89)	1024 (19.81)	0.0000
Smoking status: Current *n* (%)	3055 (17.73)	2384 (19.76)	707 (13.69)	0.0000
Age (years) mean (SE)	47.71 (0.35)	45.55 (0.35)	52.0 (0.41)	0.0000
Height (cm), mean (SE)	168.48 (0.16)	168.64 (0.17)	168.16 (0.27)	0.8006
Weight (kg), mean (SE)	83.37 (0.36)	81.97 (0.36)	86.15 (0.61)	0.0000
BMI (kg/m^2^), mean (SE)	29.29 (0.13)	28.74 (0.14)	30.38 (0.20)	0.0000
**NUTRINET SANTÉ (Ref.** [2] *)	**Total**	**Non-Consumers**	**Higher Consumers**	
Sample size, *n*	102,865 (100)	64,892 (63.08)	18,987 (18.46)	
Female *n* (%)	80,711 (78.46)	49,349 (76.05)	15,681 (82.59)	<0.001
Educational level *n* (%)				
<12 y	18,062 (17.42)	11,523 (17.75)	3276 (17.25)	<0.001
<14 y	17,921 (17.42)	11,269 (17.36)	3348 (17.63)	
>14 y	66,894 (65.02)	41,109 (64.88)	12,365 (65.12)	
Type 2 diabetes *n* (%)	1522 (1.48)	676 (1.04)	525 (2.76)	<0.001
Smoking status: Current *n* (%)	17,945 (17.44)	11,188 (17.24)	3859 (20.32)	>0.001
Age (years), mean (SE)	42.22 (0.04)	42.82 (0.06)	40.31 (0.10)	<0.001
Height (cm), mean (SE)	166.93 (0.02)	167.24 (0.03)	166.61 (0.06)	<0.001
BMI (kg/m^2^) mean (SE)	23.69 (0.01)	23.29 (0.02)	24.96 (0.04)	<0.001

Note: PIR, Poverty-to-Income ratio; BMI, body mass index, kg/m^2^. * See Table 1 in [2] for more detailed data and statistics.

## Data Availability

All US databases used are publicly available and can be downloaded from websites of the Centers for Disease Control and Prevention (CDC) and the National Center for Health Statistics (NCHS). The databases are NHANES 2011-16 Analytic Guidelines, 2011–2016, available at http://wwwn.cdc.gov/Nchs/data/nhanes/analyticguidelines/11-16-analytic-guidelines.pdf (accessed on 6 August 2025), and NHANES 2015–201: Sample Design and Estimation Procedures, available at http://stacks.cdc.gov/view/cdc/88305 (accessed on 6 August 2025). The FNDDS nutrient composition data are at FoodData Central at https://www.ars.usda.gov/northeast-area/beltsville-md-bhnrc/beltsville-human-nutrition-research-center/food-surveys-research-group/docs/fndds/ (accessed on 6 August 2025). NutriNet Santé nutrient composition and dietary intakes data are not publicly available.

## Supplementary Materials

The following supporting information can be downloaded at: https://www.mdpi.com/article/10.3390/nu17162581/s1, Table S1: Drewnowski and Fulgoni; Table S2: Comparisons of diet quality metrics of LCS consumers and non-consumers in NHANES 2011-18.
