# Supplementary material for: Should Atypical and Non-Representative Studies Such as NutriNet Santé Be Used to Drive Public Health Policy?
Source: Nutrients. 2025 Aug 8;17(16):2581. doi: 10.3390/nu17162581 (PMC12389494; doi:10.3390/nu17162581)
Supplement: Supplementary file 1 [file nutrients-17-02581-s001.zip › nutrients-3783615-supplementary.pdf]

Supplementary Table S1 Drewnowski and Fulgoni

Foodcode	Description	lcs type	kcal	add sugars	tsug
11422100	yogurt, vanilla, low fat milk, light	food	85.95	0.00	5.43
11424000	yogurt, vanilla, nonfat milk, light	food	43.83	0.01	7.50
11432500	yogurt, fruit, low fat milk, light	food	105.09	0.00	2.90
11433500	yogurt, fruit, nonfat milk, light	food	48.52	0.00	6.78
11460400	yogurt, frozen, chocolate, nonfat milk, with low-calorie sweetener	food	107.05	0.00	12.75
11460410	yogurt, frozen, flavors other than chocolate, nonfat milk, with low-calorie sweetener	food	114.80	0.00	17.88
11514300	cocoa with nonfat dry milk and low calorie sweetener, mixture, water added	beverage	28.04	0.00	3.11
11514500	cocoa, whey, and low calorie sweetener, mixture, fortified, water added	beverage	51.55	0.00	4.86
11516000	cocoa, whey, and low-calorie sweetener mixture, lowfat milk added	beverage	50.71	0.00	6.13
11518000	milk beverage with nonfat dry milk and low calorie sweetener, water added, chocolate	beverage	34.20	0.00	3.40
11613000	instant breakfast, powder, sweetened with low calorie sweetener, milk added	beverage	65.65	0.00	7.72
11623000	meal supplement or replacement, commercially prepared, ready-to-drink	beverage	70.04	6.12	10.89
11830110	cocoa powder with nonfat dry milk and low calorie sweetener, dry mix, not reconstituted	beverage	350.28	0.00	41.18
11830120	cocoa, whey, and low calorie sweetener, fortified, dry mix, not reconstituted	beverage	362.07	0.00	55.60
11830170	cocoa (or chocolate) flavored beverage powder with low-calorie sweetener, dry mix, not reconstituted	beverage	367.79	0.00	35.48
11830500	milk beverage, powder, with nonfat dry milk and low calorie sweetener, dry mix, not reconstituted, chocolate	beverage	300.00	0.00	40.46
11830810	instant breakfast, powder, sweetened with low calorie sweetener, not reconstituted	beverage	357.02	0.00	35.60
13160100	milk dessert,fzn,lowfat,w/low cal sweet,not choc	food	116.83	1.72	16.38
13160160	fat free ice cream, no sugar added, flavors other than chocolate	food	113.42	0.00	18.88
13160600	milk dessert, froz, w/ low cal sweetener, not choc	food	142.06	0.43	18.23
13161600	fudgesicle, light	food	95.08	0.00	4.73
13161630	light ice cream, bar or stick, with low-calorie sweetener, chocolate coated	food	221.85	0.00	10.66
13210250	pudding, chocolate, low calorie, containing artificial sweetener, ns as to from dry mix or ready-to-eat	food	83.31	7.73	11.45
13210290	pudding, flavors other than chocolate, low calorie, containing artificial sweetener, ns as to from dry mix or ready-to-eat	food	80.03	4.99	11.12
13220210	pudding, flavors other than chocolate, made from dry mix, sugar free	food	65.97	0.00	4.78
13220220	pudding, chocolate, made from dry mix, sugar free	food	66.84	0.00	4.67
13230120	pudding, flavors other than chocolate, ready-to-eat, sugar free	food	87.59	11.12	13.87
13230140	pudding, chocolate, ready-to-eat, sugar free	food	89.34	10.39	14.62
28401200	gelatin drink, powder, flavored, with low-calorie sweetener, reconstituted	beverage	19.99	0.00	0.00
53123500	cake, shortcake, with whipped topping and fruit, diet	food	192.55	17.07	20.91
57101020	all-bran with extra fiber	food	182.23	0.00	4.21
57206700	cereal (general mills fiber one)	food	201.96	0.00	0.72
63101140	applesauce, stewed apples, sweetened with low calorie sweetener	food	42.72	0.00	9.51

63420200	fruit juice bar, frozen, sweetened with low calorie sweetener, flavors other than orange	food	44.43	8.82	10.69
64105400	cranberry juice, 100%, not a blend	food	47.31	0.00	8.93
91106000	sugar substitute, sugar-aspartame blend, dry powder	tabletop	18.94	4.92	4.92
91106010	sugar substitute and sugar blend	tabletop	4.65	1.13	1.15
91107000	sugar substitute, sucralose, powder	tabletop	3.42	0.00	0.82
91108000	sugar substitute, stevia, powder	tabletop	0.14	0.00	0.00
91108010	sugar substitute, stevia, liquid	tabletop	0.42	0.00	0.00
91108020	sugar substitute, monk fruit, powder	tabletop	0.00	0.00	0.00
91200000	sugar substitute, powder, nfs	tabletop	4.46	0.00	1.03
91200005	sugar substitute, liquid, nfs	tabletop	0.42	0.00	0.00
91200020	sugar substitute, saccharin-based, dry powder	tabletop	2.08	0.00	0.17
91200030	brown sugar substitute, saccharin-based, dry powder	tabletop	1.17	0.00	0.20
91200040	sugar substitute, saccharin, powder	tabletop	3.20	0.00	0.51
91200110	sugar substitute, saccharin, liquid	tabletop	0.07	0.00	0.02
91201010	sugar substitute, aspartame, powder	tabletop	4.01	0.00	0.83
91351010	syrup, dietetic	food	42.66	0.00	0.97
91405000	jelly, sugar free, all flavors	food	120.35	0.00	28.24
91406000	jam, preserve, marmalade, sugar free, all flavors	food	111.67	0.00	34.79
91501020	gelatin dessert with fruit	food	64.72	8.53	13.67
91510100	gelatin powder, dietetic, sweetened with low calorie sweetener, dry	beverage	262.12	0.00	0.00
91511010	gelatin dessert, sugar free	food	4.81	0.00	0.00
91511020	gelatin dessert, sugar free, with fruit	food	35.34	0.00	5.42
91511030	gelatin dessert, dietetic, with whipped topping, sweetened with low calorie sweetener	food	24.50	0.00	0.79
91511050	gelatin dessert, dietetic, with cream cheese, sweetened with low calorie sweetener	food	49.73	0.00	0.44
91511060	gelatin dessert, dietetic, with sour cream, sweetened with low calorie sweetener	food	46.65	0.00	0.05
91511070	gelatin dessert, dietetic, with fruit and sour cream, sweetened with low calorie sweetener	food	71.52	0.00	4.83
91511080	gelatin dessert, dietetic, with fruit and cream cheese, sweetened with low calorie sweetener	food	57.72	0.00	9.06
91511090	gelatin dessert, dietetic, with fruits and vegetables, sweetened with low calorie sweetener	food	47.90	0.00	4.81
91511100	gelatin salad, dietetic, with vegetables, sweetened with low calorie sweetener	food	6.27	0.00	0.50
91511110	gelatin dessert, dietetic, with fruit and whipped topping, sweetened with low calorie sweetener	food	39.97	0.00	5.63
91611100	popsicle, no sugar added	food	24.07	1.51	2.39
92121030	coffee, mocha, instant, pre-lightened and pre-sweetened with low calorie sweetener, reconstituted	beverage	11.44	1.09	1.09
92121040	coffee, instant, pre-lightened and pre-sweetened with low calorie sweetener, reconstituted	beverage	20.68	2.13	2.13
92121041	coffee, instant, decaffeinated, pre-lightened and pre-sweetened with low calorie sweetener, reconstituted	beverage	15.95	0.59	0.60
92121050	coffee, mocha, instant, decaffeinated, pre-lightened and pre-sweetened with low calorie sweetener, reconstituted	beverage	24.00	2.56	2.56
92130005	coffee, pre-lightened and pre-sweetened with low calorie sweetener	beverage	16.96	1.76	1.76
92130006	coffee, decaffeinated, pre-lightened and pre-sweetened with low calorie sweetener	beverage	16.90	1.76	1.76

92130030	coffee, pre-sweetened with low calorie sweetener	beverage	4.82	0.00	0.45
92130031	coffee, decaffeinated, pre-sweetened with low calorie sweetener	beverage	4.75	0.00	0.45
92192040	coffee, mocha, instant, decaffeinated, pre-lightened and pre-sweetend with low calorie sweetener, not reconstituted	beverage	486.30	34.99	34.99
92193020	coffee, instant, pre-lightened and pre-sweetened with low calorie sweetener, not reconstituted	beverage	513.19	59.01	59.01
92301080	tea, ns as to type, presweetened with low calorie sweetener	beverage	2.60	0.00	0.33
92301180	tea, ns as to type, decaffeinated, presweetened with low calorie sweetener	beverage	2.54	0.00	0.34
92302300	tea, leaf, presweetened with low calorie sweetener	beverage	2.56	0.00	0.22
92302700	tea, leaf, decaffeinated, presweetened with low calorie sweetener	beverage	2.54	0.00	0.33
92305090	tea, iced, instant, black, pre-sweetened with low calorie sweetener	beverage	2.00	0.00	0.01
92305110	tea, iced, instant, black, decaffeinated, pre-sweetened with low calorie sweetener	beverage	2.33	0.00	0.01
92305920	tea, iced, instant, green, pre-sweetened with low calorie sweetener	beverage	0.00	0.00	0.93
92306030	tea, herbal, presweetened with low calorie sweetener	beverage	2.52	0.00	0.33
92308010	tea, iced, brewed, black, pre-sweetened with low calorie sweetener	beverage	2.39	0.00	0.33
92308040	tea, iced, brewed, black, decaffeinated, pre-sweetened with low calorie sweetener	beverage	2.47	0.00	0.33
92308510	tea, iced, brewed, green, pre-sweetened with low calorie sweetener	beverage	2.40	0.00	0.33
92308540	tea, iced, brewed, green, decaffeinated, pre-sweetened with low calorie sweetener	beverage	1.40	0.00	0.33
92400100	soft drink, nfs, diet	beverage	1.59	0.00	0.00
92410250	carbonated water, sweetened, with low-calorie or no-calorie sweetener	beverage	0.00	0.00	0.00
92410320	soft drink, cola, diet	beverage	1.70	0.00	0.00
92410350	soft drink, cola, decaffeinated, diet	beverage	1.04	0.00	0.00
92410370	soft drink, pepper type, diet	beverage	1.78	0.00	0.00
92410400	soft drink, pepper type, decaffeinated, diet	beverage	1.05	0.00	0.00
92410420	soft drink, cream soda, diet	beverage	0.00	0.00	0.00
92410520	soft drink, fruit flavored, diet, caffeine free	beverage	0.00	0.00	0.00
92410560	soft drink, fruit flavored, caffeine containing, diet	beverage	0.00	0.00	0.00
92410620	soft drink, ginger ale, diet	beverage	0.00	0.00	0.00
92410720	soft drink, root beer, diet	beverage	0.00	0.00	0.00
92410820	soft drink, chocolate flavored, diet	beverage	1.19	0.00	0.00
92411610	soft drink, cola, fruit or vanilla flavored, diet	beverage	1.77	0.00	0.00
92511260	orange-cranberry juice drink	beverage	47.35	8.11	12.88
92520410	fruit drink, low calorie	beverage	18.96	8.11	8.11
92520810	grape drink, low calorie	beverage	18.25	8.11	8.11
92520910	lemonade, low calorie	beverage	3.75	8.11	8.11
92541040	lemonade-flavored drink, made from powdered mix, low calorie	beverage	2.09	8.74	8.74
92550110	cranberry juice drink, with high vitamin c, light	beverage	19.06	2.60	4.54
92550210	cranberry-apple juice drink, low calorie, with vitamin c added	beverage	19.02	2.60	4.36
92550300	grapefruit juice drink, low calorie, with vitamin c added	beverage	13.61	2.60	3.50

92550400	vegetable and fruit juice drink, with high vitamin c, diet	beverage	4.02	0.00	0.63
92550610	fruit flavored drink, with high vitamin c, diet	beverage	9.54	0.16	1.76
92550620	fruit flavored drink, diet	beverage	3.14	0.00	0.00
92551600	citrus juice drink, low calorie	beverage	29.94	0.00	7.66
92551700	juice drink, low calorie	beverage	19.91	0.00	4.76
92552000	fruit flavored drink, with high vitamin c, powdered, reconstituted, diet	beverage	4.66	0.00	0.00
92552010	fruit flavored drink, powdered, reconstituted, diet	beverage	1.53	0.00	0.00
92552100	orange-cranberry juice drink, low calorie, with vitamin c added	beverage	11.81	2.60	2.87
92553000	fruit-flavored thirst quencher beverage, low calorie	beverage	10.98	0.00	0.00
92565000	fruit-flavored sports drink or thirst quencher beverage, low calorie	beverage	4.03	0.63	0.65
92565100	gatorade g2 thirst quencher sports drink, low calorie	beverage	7.99	1.30	1.30
92565200	powerade zero sports drink, low calorie	beverage	0.00	0.00	0.00
92650005	red bull energy drink, sugar-free	beverage	5.00	0.00	0.00
92650210	mountain dew amp energy drink, sugar-free	beverage	1.99	0.00	0.00
92650705	rockstar energy drink, sugar-free	beverage	4.00	0.00	0.00
92650805	vault zero energy drink	beverage	1.07	0.00	0.00
92741000	fruit-flavored drink, non-carbonated, made from low calorie powdered mix	beverage	1.71	0.00	0.00
92900200	fruit flavored drink, powdered, not reconstituted, diet	beverage	217.12	0.00	0.00
94100200	water, bottled, sweetened, with low calorie sweetener	beverage	1.00	0.00	0.00
95106010	nutritional drink or shake, ready-to-drink, light (muscle milk)	beverage	36.31	0.00	0.28
95110000	nutritional drink or shake, ready-to-drink (slim fast)	beverage	57.01	1.39	5.95
95110010	nutritional drink or shake, ready-to-drink, sugar free (slim fast)	beverage	56.94	0.00	5.95
95110020	nutritional drink or shake, high protein, ready-to-drink (slim fast)	beverage	58.06	0.00	0.60
95120010	nutritional drink or shake, high protein, ready-to-drink, nfs	beverage	57.97	0.00	0.60
95312400	energy drink, low calorie (monster)	beverage	5.01	1.39	1.39
95312500	energy drink, sugar free (mountain dew amp)	beverage	2.02	0.00	0.00
95312550	energy drink, sugar free (no fear)	beverage	3.96	0.00	0.00
95312600	energy drink, sugar-free (red bull)	beverage	5.02	0.00	0.00
95312700	energy drink, sugar free (rockstar)	beverage	3.97	0.00	0.00
95312800	energy drink, sugar free (vault)	beverage	0.83	0.00	0.00
95312900	energy drink (xs)	beverage	5.00	0.00	0.00
95313200	energy drink, sugar free	beverage	4.56	0.00	0.00
95322200	sports drink, low calorie (gatorade g2)	beverage	8.00	1.30	1.30
95322500	sports drink, low calorie (powerade zero)	beverage	0.00	0.00	0.00
95323000	sports drink, low calorie	beverage	7.95	1.30	1.30
95341000	fuze slenderize fortified low calorie fruit juice beverage	beverage	2.33	0.00	0.00

Supplementary Table S2: Comparisons of diet quality metrics of LCS consumers and non-consumers in NHANES 2011-18.

NHANES 2011-18	All n=17,252		Non-consumers n=12,085		Consumers n=5,168		
	Mean	SE	Mean	SE	Mean	SE	P value
HEI-2020 total score (100 pt)	53.78	0.3	53.18	0.35	54.98	0.40	0.0001
Total vegetables	3.27	0.02	3.20	0.03	3.41	0.03	0.0000
Greens and beans	2.06	0.03	2.05	0.04	2.07	0.05	0.7126
Total fruit	2.30	0.04	2.26	0.04	2.39	0.04	0.0157
Whole fruit	2.50	0.05	2.41	0.06	2.68	0.05	0.0000
Whole grains	2.90	0.05	2.75	0.07	3.19	0.07	0.0000
Dairy	5.28	0.05	5.19	0.06	5.45	0.08	0.0030
Total protein foods	4.51	0.01	4.47	0.02	4.59	0.02	0.0000
Seafood and plant protein	2.95	0.03	2.88	0.04	3.08	0.05	0.0012
Fatty acid ratio	4.97	0.05	4.93	0.05	5.06	0.08	0.1674
Sodium	4.03	0.05	4.28	0.05	3.53	0.07	0.0000
Refined grains	6.29	0.05	6.27	0.06	6.31	0.09	0.7403
Saturated fat	5.78	0.05	5.93	0.05	5.48	0.08	0.0000
Added sugar	6.95	0.06	6.55	0.07	7.74	0.07	0.0000
